# Psychedelic substitution: altered substance use patterns following psychedelic use in a global survey

**DOI:** 10.3389/fpsyt.2024.1349565

**Published:** 2024-02-22

**Authors:** Nicolas G. Glynos, Jacob S. Aday, Daniel Kruger, Kevin F. Boehnke, Stephanie Lake, Philippe Lucas

**Affiliations:** ^1^ Anesthesiology Department, University of Michigan Medical School, Ann Arbor, MI, United States; ^2^ Chronic Pain and Fatigue Research Center, University of Michigan Medical School, Ann Abor, MI, United States; ^3^ Michigan Psychedelic Center, University of Michigan, Ann Arbor, MI, United States; ^4^ Jacobs School of Medicine and Biomedical Sciences, University at Buffalo, State University of New York, Buffalo, NY, United States; ^5^ University of California Los Angeles (UCLA) Center for Cannabis and Cannabinoids, Jane and Terry Semel Institute for Neuroscience and Human Behavior, University of California, Los Angeles, Los Angeles, CA, United States; ^6^ Department of Psychiatry and Biobehavioral Sciences, David Geffen School of Medicine, University of California, Los Angeles, Los Angeles, CA, United States; ^7^ SABI Mind, Calgary, AB, Canada

**Keywords:** psychedelics, psilocybin, MDMA, survey, SUD, substance use

## Abstract

**Introduction:**

Recent research suggests that psychedelics may have potential for the treatment of various substance use disorders. However, most studies to date have been limited by small sample sizes and neglecting to include non-North American and European populations.

**Methods:**

We conducted a global, cross-sectional online survey of adults (n = 5,268, 47.2% women) self-reporting past or current psychedelic use and investigated whether psychedelic use was associated with changes in use of other substances.

**Results:**

Nearly three-quarters (70.9%; n = 3,737/5,268) reported ceasing or decreasing use of one or more non-psychedelic substances after naturalistic psychedelic use. Among those with previous use, 60.6% (n = 2,634/4,344) decreased alcohol use, 55.7% (n = 1,223/2,197) decreased antidepressant use, and 54.2% (n = 767/1,415) decreased use of cocaine/crack. Over a quarter of the sample indicated that their decrease in substance use persisted for 26 weeks or more following use of a psychedelic. Factors associated with decreased use included a motivation to either decrease one’s substance use or self-treat a medical condition. Importantly, 19.8% of respondents also reported increased or initiated use of one or more other substances after psychedelic use, with illicit opioids (14.7%; n = 86/584) and cannabis (13.3%; n = 540/4,064) having the highest proportions. Factors associated with increased substance use included having a higher income and residing in Canada or the US.

**Discussion:**

Although limited by cross-sectional study design, this large observational study will help inform future studies aiming to investigate the relationship between substance use patterns and psychedelic use.

## Introduction

There is a growing body of clinical and observational evidence suggesting the potential therapeutic benefits of psychedelic substances and psychedelic-assisted therapies (1, 2). These substances include “classical” psychedelics such as lysergic acid diethylamide (LSD), *N*,*N*-dimethyltryptamine (DMT), psilocybin, and mescaline, as well as “atypical” psychedelics such as 3,4-methylenedioxymethamphetamine (MDMA), ketamine, nitrous oxide, and ibogaine (3). An increasing number of clinical trials with psychedelics over the past two decades have demonstrated promising results for the treatment of depression (4–6), anxiety (5, 7), post-traumatic stress disorder (8), and substance use disorders including tobacco addiction (9) and alcohol use disorder (10). Observational studies also suggest that psychedelics may improve symptoms of these conditions in naturalistic (11–15), clinical (16, 17), and retreat settings (18, 19). In tandem, psychedelic use across the globe continues to increase (20), and policy changes are continually being proposed or enacted to legalize and/or decriminalize possession and use of these substances (21).

This resurgence of interest in psychedelics is occurring amidst a time when mental health disorders, including substance use disorders (SUDs), continue to present a substantial economic and health burden across the globe (22, 23). In the mid-20th century, SUDs were some of the first indications to be investigated by early psychedelic researchers, and this potential application continues to be a focus of the contemporary psychedelic renaissance (24). Indeed, LSD received considerable interest as a treatment for alcohol use disorder from 1950-1970, with a meta-analysis of 6 randomized-controlled trials within that time (n = 536 total) showing that LSD in conjunction with various alcoholism treatment programs was associated with a decrease in alcohol misuse for up to three months (25). Modern clinical trials with psilocybin have supported these findings, with a larger double-blind, randomized, placebo-controlled trial (n = 95) finding significant reductions in percentage of heavy drinking days and mean daily alcohol consumption during the 32-week follow-up period in the psilocybin group relative to the active control diphenhydramine (10). Psilocybin therapy also shows preliminary efficacy in the treatment of tobacco addiction, with a pilot study showing that 12 out of 15 (80%) regular tobacco smokers were abstinent at 6-month follow up following moderate (20 mg/70kg) and high (30mg/70kg) doses of psilocybin (26). A long-term follow up (16–57 months) with these participants revealed that 60% remained smoking abstinent following the first psilocybin dosing session (9).

Naturalistic use of psychedelics, which occurs outside of formal treatment settings and does not often include structured therapeutic supports typically involved in clinical trials, has also been associated with reduced use of various substances such as alcohol, tobacco, opioids, and stimulants (15, 27–33). We recently conducted a survey of 1,435 adults in the US and found that many reported decreased use of alcohol (37.2%; n = 534/1,435), tobacco/nicotine (18.3%; n = 263/1,435), and prescription opioids (7.2%; n = 104/1,435) after naturalistic psychedelic use (34). In a more recent study of 1,639 Canadian adults, we showed that 52.8% (n = 866/1,639) reported decreasing their use of one or more non-psychedelic substances after naturalistic psychedelic use, including alcohol (43.7%; n = 651/1,488), antidepressants (42.5%; n = 272/640), and cocaine (42.4%; n = 200/471) (35). These studies are limited, however, in that they are restricted to residents of North America, and little is known regarding the effects of naturalistic psychedelic use on global patterns of substance use.

Thus, in the current study we expanded upon our previous research with a larger, global sample of adults who reported past or current psychedelic use. We assessed the perceived effects of psychedelic use on reported changes in non-psychedelic substance use and explored associations between demographic and psychedelic use characteristics with self-reported changes in substance use. In line with previous findings, we hypothesized that changes in other substance use perceived to result from psychedelic use will be commonly reported among those with a history of other substance use, with a majority reporting decreased substance use. In addition, we hypothesized that psilocybin would be the psychedelic most commonly associated with decreased use of any non-psychedelic substance.

## Methods

The data presented here were collected from the Global Psychedelic Survey, a large self-reported cross-sectional survey that was distributed between May 19 and June 2, 2023 via online psychedelic organizations including the Multidisciplinary Association for Psychedelic Studies (MAPS) and MAPS Canada, The Psychedelic Association of Canada, OPEN Foundation, Mind Medicine Australia, International Center for Ethnobotanical Education, Research and Service (ICEERS), and social media. This survey gathered data on psychedelic use trends among English-speaking adults 21 years of age or older across the globe. The survey targeted 11 substances with psychedelic-like properties including the “classic” psychedelics ayahuasca, DMT/5-MeO-DMT (*N*,*N*-dimethyltryptamine/5-methoxy-*N*,*N*-dimethyltryptamine), LSD/acid, mescaline, and psilocybin, as well as several “atypical” psychedelics including 2C-B (4-Bromo-2,5-dimethoxyphenethylamine), iboga/ibogaine, ketamine, MDMA/MDA (3,4-methylenedioxymethamphetamine/3,4-methylenedioxyamphetamine), nitrous oxide, and *Salvia divinorum*. The survey was co-sponsored by MAPS, Mind Medicine Australia, SABI Mind, and Tiny. The study was reviewed by Advarra (protocol # Pro00071490) to help ensure that the rights and welfare of research participants were protected and that the research study was carried out in an ethical manner. However, the IRB only oversaw Canadian subjects, determining that this international study was otherwise exempt from IRB oversight in other jurisdictions under Department of Health and Human Services regulation 45 CFR 46.104(d) (2).

### Sample

All participants were English literate adults (≥21 years) who self-reported past or current use of one or more of the 11 psychedelics included in the survey. Informed consent to participate was gathered online as part of the survey, and all responses were collected anonymously. Upon survey completion, participants were given the option to provide their email address to be entered into a drawing for one of three $500 Amazon gift cards. All email addresses were stored separately from the study data and were deleted prior to data analysis to ensure confidentiality and anonymity of participants. Data gathering was conducted via the Quantified Citizen website and app, which are both Personal Information Protection and Electronic Documents Act (PIPEDA) and Health Insurance Portability and Accountability Act (HIPAA) compliant.

### Measures

A complete list of the questions in the survey is provided as a **Supplementary Material**. In addition to addressing demographic characteristics such as gender, country of residence, age, education, and income, the survey also included questions about various aspects of psychedelic use such as number (i.e. variety) of psychedelics used, typical dosages consumed (microdoses only, macrodoses only, both micro and macro doses), and motivations for using psychedelics with possible responses including: *to treat a medical condition, to reduce use of another substance, general well-being, personal growth/self-exploration, religious/spiritual development, recreational*, or *none of the above*. In addition, we assessed lifetime use of non-psychedelic substances and medications, including alcohol, cannabis, nicotine/tobacco, antidepressants, benzodiazepines (e.g., Valium, Ativan, etc.), prescription or non-prescription amphetamines (e.g., Ritalin, Adderall, speed, crystal meth), cocaine/crack, prescription opioids (e.g., Fentanyl, Oxycodone, Hydromorphone, etc.), and illicit opioids (e.g., heroin, Fentayl, Oxycodone, Hydromorphone, etc.).

### Changes in substance use

We asked participants if their use of any of the non-psychedelic substances changed “as a result” of their psychedelic use via 9 questions (one for each non-psychedelic substance of interest listed above). Respondents who endorsed lifetime use of a non-psychedelic substance through a separate question at the survey outset were presented with the corresponding change in use question for each endorsed substance later in the survey. The exception was antidepressants, for which the changes in use question was presented to all respondents, as lifetime use was not assessed with the other substances as the survey outset. For each of the 9 categories of non-psychedelic substances, participants indicated whether their use of psychedelics resulted in ceased use, decreased use, no change in use, initiation of use, increased use, or an option for not having used that substance. We included the latter category due to the inclusion of antidepressants (asked to all participants) and as we expected some respondents with lifetime use of a substance to not identify with any other listed response option (e.g., cases of one-time use many years before survey completion). Participants who reported ceasing or decreasing substance use as a result of naturalistic psychedelic use then identified the psychedelic substance that was particularly impactful for ceasing or decreasing the use of other substances. We also asked these participants how psychedelics helped them cease or decrease use of other substances in a “select all that apply” format with possible options including: *they made me feel more connected to myself, they changed my relationship with/or perspective on other substances, they made me less anxious or depressed, they helped me resolve past trauma, they made me feel more connected with nature, they made me feel more connected with others, they made me feel more connected with spirit, they reduced cravings/urges*, or *they reduced withdrawal.* Finally, we asked participants who reported ceased or decreased use how long the decrease in substance use typically persisted after using psychedelics. Possible response options included: *Less than one week, 1 – 4 weeks, 5 – 11 weeks, 12 – 26 weeks, more than 26 weeks*, or *no set pattern.*

### Data cleaning

Prior to analysis, data were cleaned and prepared by Precision Analytics. In addition to removing duplicate responses, data were excluded if the participant’s reported age was either missing or less than 21 years, or if they did not report naturalistic use of one of the 11 psychedelic substances in their lifetime. This resulted in a dataset of 6,379 valid responses for the Global Psychedelic Survey. The current report focuses only on those who were eligible to respond to at least one question related to change in non-psychedelic substance use and provided a valid response aside from the “*NA – I don’t use this substance*” option. The final analytic sample includes data from 5,268 respondents, which are included in the subsequent analyses.

### Statistical analysis

We first characterized the sample via descriptive statistics. We then sub-grouped participants by whether they reported ceased/decreased use of any substance (ceased/decreased use vs. no ceased/decreased use). We compared associations between these two sub-groupings and socio-demographic characteristics using chi-square tests and the Cramer V statistic for categorical variables, and independent sample t-tests and the Cohen d statistic for continuous variables. The details about how psychedelics contributed to ceased or decreased use of other substances are reported as proportions among those who reported ceased or decreased use. To identify potential predictors of changed substance use, we conducted binary logistic regression analyses for two separate outcome variables: one included only those who reported ceased or decreased substance use, and another included only those who reported increased or initiated use. Predictors in the model included: age, gender identity (female, other gender [gender fluid, transgender, a different gender] vs. male), education level (scored continuously from: less than high school, high school degree or equivalent, technical or non-university degree, university degree [Bachelor’s or equivalent], graduate degree [MA, MSc, etc.], doctorate or professional degree [JD, MD, PhD, etc.]), income (scored continuously from: very low income/well below average, low income/below average, middle income/about average, high income/above average, very high income/well above average), race/ethnicity (White/Caucasian vs. other), region of residence (Europe/United Kingdom, Canada/United States, Australia/New Zealand vs. other), number of psychedelics used (up to 12), type of psychedelic dose(s) consumed (both macro and microdoses, only microdoses, vs. only macrodoses), motivations for psychedelic use (medical, recreational, to reduce other substance use; all yes vs. no). Significance was set at α = 0.05 (two-sided), and all analyses were conducted using SPSS version 29.

## Results

The final study sample consisted of n = 5,268 adults, which was 51.1% male and 41.1 ± 12.5 years of age on average. Overall, n = 3,737 (70.9%) reported ceased or decreased use of one or more substances as a result of psychedelic use, with n = 1,531 (29.1%) reporting not ceasing or decreasing use of any substances. A higher proportion of those who ceased or decreased their substance use were male (p = .01), were younger (p <.001), had used a larger variety of psychedelics (p <.001), consumed both micro and macro doses (p <.001), were less educated (p <.001), and had lower incomes (p <.01) (**Table 1**). Compared to women, a higher proportion of men reported lifetime use of all substances except benzodiazepines.

**Table 1:** Socio-demographics sub-grouped by how use of other substances changed following psychedelic use.

Descriptive	Ceased or decreased use (n=3,737)	No ceased or decreased use (n=1,531)	*t* or χ2	P value
**Gender**			12.46	.014
Women	45.7% (1,709)	50.6% (775)		
Men	51.1% (1,908)	46% (705)		
Non-Binary	2.2% (81)	2.5% (39)		
Other	0.8% (30)	0.6% (9)		
Prefer not to say	0.2% (9)	0.2% (3)		
**Region of residence**			2.38	0.795
Asia Pacific	14.6% (546)	14.6% (224)		
Central Asia, Middle East, Africa	0.9% (34)	1.2% (19)		
Europe	11.6% (434)	11.8% (180)		
Latin America	2.8% (104)	2.4% (36)		
North America	70.1% (2,618)	70% (1,072)		
Other	< 0.1% (1)	0		
**Age in years (M, SD, range)**	41.1, 12.5, 21 - 90	46.9, 14.8, 21 - 87	-14.26	<.001
21-24	6.2% (231)	4.4% (67)		
25-34	30.2% (1,127)	19.5% (299)		
35-44	30.0% (1,121)	24.0% 368)		
45-54	17.5% (655)	20.5% (314)		
55-64	9.5% (356)	15.9% (243)		
>65	6.6% (247)	15.7% (240)		
**Number of psychedelics used** **(M, SD, range)**	5.0, 2.4, 1 - 12	3.9, 2.2, 1 - 12	15.18	<.001
**Dosage used**			273.62	<.001
Microdose only	1.5% (56)	2.7% (41)		
Macrodose only	19.3% (723)	40.5% (620)		
Both micro and macro doses	79.1% (2,957)	56.8% (869)		
Missing	<0.1% (1)	<0.1% (1)		
**Education**			50.38	<.001
Less than high school	1.9% (72)	1.0% (16)		
High school or equivalent	13.6% (510)	10.5% (161)		
Technical degree	14.4% (538)	10.8% (165)		
Bachelor’s degree or equivalent	33.8% (1,263)	32.1% (491)		
Graduate degree	24.8% (927)	30.3% (464)		
Doctoral/professional degree	11.4% (427)	15.3% (234)		
**Income**			13.60	<.01
Very low	5.4% (202)	4.0% (61)		
Low	16.1% (603)	14.2% (217)		
Middle	43.7% (1,633)	43.6% (667)		
High	27.6% (1,031)	31.6% (484)		
Very high	7.2% (268)	6.7% (102)		

**Figure 1:**
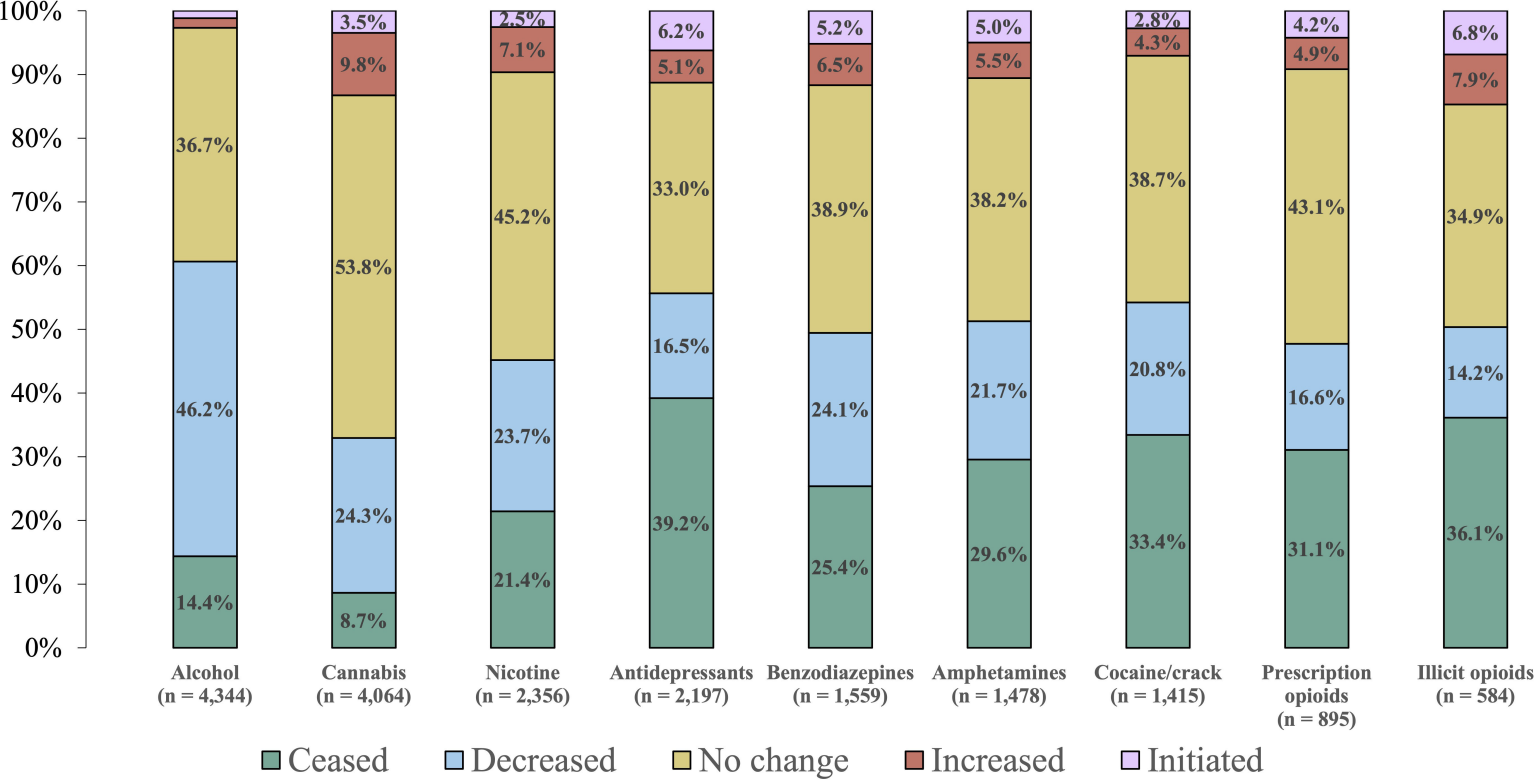
Self-reported changes in substance use following psychedelic use. The number of participants who reported past or current use of each of the substances is listed below each substance. Proportions for each category are listed in their respective locations, and values less than 2.0% are not shown.

Overall, more than half of participants reported ceased or decreased use of several substances they endorsed previously using, including alcohol (60.6%; n = 2,634/4,344), antidepressants (55.7%; n = 1,223/2,197), cocaine/crack (54.2%; n = 767/1,415), amphetamines (51.3%; n = 758/1,478), and non-prescription opioids (50.3%; n = 294/584). Cannabis had the lowest proportion of ceased or decreased use (32.9%; n = 1,339/4,064) (**Figure 1**). In contrast, reports of increasing or initiating use of other substances were lower globally, with 19.8% reporting any increased or initiated use, with illicit opioids (14.7%; n = 86/584), cannabis (13.3%; n = 540/4,084), benzodiazepines (11.7%; n = 182/1,559), antidepressants (11.3%; n = 248/2,197), amphetamines (10.6%; n = 156/1,478), and tobacco/nicotine (9.6%; n = 227/2,356) being the most endorsed substances for increased/initiated use. Alcohol had the lowest proportion of reported increased or initiated use (2.7%; n = 117/4,344) (**Figure 1**). A higher proportion of participants residing in North America reported increased or initiated use for all nine of the non-psychedelic substance categories relative to those who reside in other regions of the globe (**Supplementary Figure 1**).

Psilocybin was reported as being the most impactful psychedelic for decreasing or ceasing substance use (44.7%; n = 1,671/3,737), followed by LSD (13.2%; n = 493/3,737) and ayahuasca (9.3%; n = 346/3,737) (**Table 2**). Among those who reported psilocybin as most impactful, 88.7% (n = 1,483/1,671) reported using more than one psychedelic in their lifetime. Nearly half of participants (42.1%; n = 1,572/3,737) reported that their decrease in substance use did not follow a set pattern or that it depends on the substance and circumstances, and over a quarter (26.4%; n = 986/3,737) reported that the resulting decrease in substance use typically lasts for more than 26 weeks. The most often self-reported reasons for how psychedelics helped to decrease use of other substances were: *feeling more connected with self* (70.2%; n = 2,620/3,730), *changing relationship with/perspective on other substances* (60.3%; n = 2,248/3,730), and *feeling more connected with nature* (56.2%; n = 2,098/3,730), *spirit* (54.8%; n = 2,044/3,730), and *others* (54.2%; n = 2,023/3,730).

**Table 2:** Details about psychedelics and impacts on substance use among those who reported ceased or decreased use.

	n = 3,737	% of total
Which of the following psychedelics did you find particularly impactful in ceasing or decreasing your use of other substances?		
Psilocybin	1,671	44.7
LSD/Acid	493	13.2
Ayahuasca	346	9.3
Ketamine	248	6.6
MDMA/MDA	233	6.2
DMT/5-MeO-DMT	155	4.1
Mescaline	77	2.1
Iboga/Ibogaine	66	1.8
Nitrous Oxide	46	1.2
*Salvia divinorum*	44	1.2
2C-B	38	1.0
None of the above	315	8.4
Missing	5	0.1
How long does the decrease in substance use typically persist after using psychedelics?		
Less than one week	192	5.1
1 – 4 weeks	439	11.7
5 – 11 weeks	297	7.9
12 – 26 weeks	244	6.5
More than 26 weeks	986	26.4
No set pattern	1,572	42.1
Missing	7	0.2
How many substances have you ceased or decreased use of as a result of your psychedelic use?		
1-3	2,968	79.4
4-6	629	16.8
7-9	140	3.7
How have psychedelics helped you to cease or decrease the use of other substances?		
They made me feel more connected to myself	2,620	70.2
They changed my relationship with/or perspective on other substances	2,248	60.3
They made me feel more connected with nature	2,098	56.2
They made me feel more connected with spirit	2,044	54.8
They made me feel more connected with others	2,023	54.2
They made me less anxious or depressed	1,911	51.2
They helped me resolve past trauma	1,556	41.7
They reduced cravings/urges	1,455	39.0
They reduced withdrawal	478	12.8
None of the above	187	5.0

### Associations between changed substance use, demographics, and psychedelic use patterns

We conducted separate multivariate analyses for those who reported ceased/decreased use, as well as increased/initiated use. Significant positive associations for ceasing or decreasing use of other substances as a result of psychedelic use included: having used a larger number of psychedelics (p <.001) having used psychedelics with a motivation to reduce other substance use (p <.001), having used both micro and macro doses (p <.001), and having used psychedelics with a motivation to improve medical (mental or physical) health (p <.001). Significant negative associations for ceasing or decreasing use of other substances as a result of psychedelic use included: age (p <.001), education level (p <.01), and identifying as a gender other than male or female (p = .02) (**Table 3**). Significant positive associations for increasing or initiating use of other substances as a result of psychedelic use included: having used a larger variety of psychedelics (p <.001), income level (p <.001), residing in Canada or the US (p <.001), and having used both micro and macro doses (p = 0.03). Significant negative associations for increasing or initiating use included: age (p <.001), having used psychedelics with a recreational motivation (p <.001), having used psychedelics with a motivation to improve medical (mental or physical) health (p <.001), identifying as female gender (p <.001), and identifying as a gender other than male or female (p = .03), (**Table 4**).

**Table 3:** Predictors of factors associated with ceasing or decreasing use of other substances.

Predictor	Univariate predictions			Multivariate predictions		
	Exp(B)	(95% CI)	p	Exp(B)	(95% CI)	p
Number of psychedelics used	1.22	(1.19 - 1.26)	<.001	1.188	(1.15 - 1.23)	<.001
Reduced substance use motivation	6.645	(5.05 - 8.74)	<.001	4.602	(3.47 - 6.11)	<.001
Age	0.970	(0.97 - 0.97)	<.001	0.974	(0.97 - 0.98)	<.001
Has used micro and macro doses	2.887	(2.54 - 3.28)	<.001	1.913	(1.65 - 2.21)	<.001
Medical motivation	2.16	(1.90 - 2.46)	<.001	1.808	(1.57 - 2.09)	<.001
Education level	0.844	(0.80 - 0.89)	<.001	0.928	(0.88 - 0.98)	<.01
Other gender	0.946	(0.67 - 1.33)	0.75	0.643	(0.44 - 0.94)	0.02
White/Caucasian	0.805	(0.68 - 0.95)	0.01	0.824	(0.68 - 0.99)	0.048
Recreational motivation	1.13	(1.00 - 1.27)	0.05	0.877	(0.76 - 1.01)	0.07
Female gender	0.822	(0.73 - 0.93)	<.001	0.890	(0.78 - 1.02)	0.09
Has used only micro doses	0.55	(0.37 - 0.83)	<.01	1.320	(0.85 - 2.06)	0.22
Income level	0.916	(0.86 - 0.98)	0.01	1.032	(0.96 - 1.11)	0.40
Australia/New Zealand	0.992	(0.83 - 1.18)	0.92	1.098	(0.77 - 1.56)	0.60
Canada/United States	1.00	(0.88 - 1.14)	0.98	0.938	(0.68 - 1.29)	0.69
Europe/United Kingdom	0.986	(0.82 - 1.19)	0.88	0.926	(0.64 - 1.33)	0.68

**Table 4:** Predictors of factors associated with increasing or initiating use of other substances.

Predictor	Univariate predictions			Multivariate predictions		
	Exp(B)	(95% CI)	p	Exp(B)	(95% CI)	p
Age	0.969	(0.96 - 0.98)	<.001	0.958	(0.95 - 0.97)	<.001
Recreational motivation	0.591	(0.52 - 0.68)	<.001	0.418	(0.36 - 0.49)	<.001
Number of psychedelics used	1.166	(1.13 - 1.20)	<.001	1.176	(1.14 - 1.21)	<.001
Medical motivation	0.687	(0.60 - 0.79)	<.001	0.627	(0.54 - 0.73)	<.001
Female gender	0.624	(0.54 - 0.72)	<.001	0.689	(0.59 - 0.80)	<.001
Income level	1.223	(1.14 - 1.32)	<.001	1.173	(1.08 - 1.27)	<.001
Canada/United States	2.259	(1.90 - 2.68)	<.001	1.972	(1.34 - 2.89)	<.001
Other gender	0.747	(0.48 - 1.16)	0.20	0.591	(0.37 - 0.94)	0.03
Has used micro and macro doses	1.93	(1.62 - 2.29)	<.001	1.244	(1.02 - 1.51)	0.03
Reduced substance use motivation	1.285	(1.08 - 1.53)	<.01	1.179	(0.97 - 1.44)	0.10
Has used only micro doses	0.716	(0.40 - 1.27)	0.25	1.50	(0.80 - 2.80)	0.20
Europe/United Kingdom	0.547	(0.43 - 0.70)	<.001	0.772	(0.49 - 1.21)	0.26
White/Caucasian	1.045	(0.87 - 1.26)	0.65	1.081	(0.88 - 1.33)	0.47
Australia/New Zealand	0.407	(0.32 - 0.53)	<.001	0.85	(0.54 - 1.33)	0.47
Education level	0.967	(0.92 - 1.02)	0.22	1.02	(0.96 - 1.08)	0.54

## Discussion

Here we show that in a large, global sample of adults, 70.9% (n = 3,737) reported ceased or decreased use of one or more non-psychedelic substances following naturalistic psychedelic use. Consistent with our hypotheses, we found that a higher proportion of participants reported ceased/decreased use of other substances compared to those who reported increased/initiated use. Furthermore, we found that psilocybin was reported as the most impactful psychedelic to support decreased use of other substances. Among those who reported this, 88.7% (n = 1,483/1,671) had used more than one psychedelic in their lifetime, indicating that only a small minority endorsed psilocybin because of absence of experience with other psychedelics. This finding was unsurprising considering that psilocybin is not only widely available and commonly used in naturalistic settings (primarily via psilocybin containing mushrooms), but also among the most promising psychedelics for a variety of psychiatric disorders demonstrated by numerous clinical trials, and its designation as a ‘breakthrough therapy’ by the Food and Drug Administration (36).

We implemented separate regression models to investigate factors associated with both ceased/decreased use as well as increased/initiated use. With this lens, the most notable factors associated with changes in other substance use were related to the motivation (i.e., intention) for psychedelic use, which aligns with previous work related to the importance of “set” and “setting” for beneficial therapeutic outcomes (1, 37). In the context of substance use, these results align with previous findings that have consistently highlighted the importance of motivation for positive outcomes in the treatment of various SUDs (38–40). Indeed, we found that having a medical motivation for psychedelic use (i.e., to treat a mental or physical health condition) was positively associated with ceasing/decreased use, and negatively associated with increased/initiated use, providing strong evidence that this factor may predict decreases in substance use. Additionally, using psychedelics with the intention to reduce use of other non-psychedelic substances was significantly associated with ceasing or decreasing use of other substances, but was not associated with initiating or increasing use. Taken together, these results also align with previous work from our group indicating that having a medical motivation for psychedelic use may predict beneficial health outcomes (34, 35, 41).

Sociodemographic results indicated that men were more likely to report ceased or decreased use of other substances relative to women. However, it was also found that relative to women, a higher proportion of men reported past use of all substances except benzodiazepines. Therefore, men may have shown a higher likelihood of ceasing or decreasing use of substances relative to women simply because a higher proportion men reported past use. Furthermore, regression analysis showed that identifying as either female or other gender was negatively associated with both decreasing use of other substances and increasing use of other substances. We believe that this contradictory result could be an artifact of higher relative proportions of men reporting past substance use, and future investigations of gender differences in longitudinal studies are warranted.

The most common explanations noted by participants for reductions in substance use were *feeling more connected to self, nature, spirit*, and *others*, as well as *changing one’s relationship or perspective on other substances*. This is consistent with the observed phenomenon of increased connectedness to self, others, and the external world that has been reported following the use of psychedelics in clinical and naturalistic settings (42–46). It has also been suggested that an increased sense of connectedness following psychedelic use may be related to the occurrence of mystical experiences, emotional breakthroughs, and a sense of belonging—all of which have been shown to correlate with positive therapeutic outcomes of psychedelic-assisted therapies (47). Importantly, an improved sense of connection to self and others may be both preventative of SUD and help support and sustain effective treatment and recovery (48–50). Therefore, these changes in relationship with nature, self, and other substances may help explain the persistence of reported decreases in substance use, with over a quarter of the population (26.4%; n = 986/3,737) indicating that the decreased substance use following naturalistic psychedelic use persisted for more than 26 weeks. Considering the growing body of research demonstrating the importance of social connectedness to overall physical health and psychological well-being (51, 52), it will be important for future studies to investigate the role that psychedelics may have in improving social connectedness and thereby mitigating potential health risks.

In addition to the large proportion of participants who reported ceased or decreased use of substances, a notable proportion (19.8%) also indicated increased or initiated use of one or more substances as a result of their psychedelic use. Illicit opioids (14.7%; n = 86/584) and cannabis (13.3%; n = 540/4,064) showed the highest overall rates of increased/initiated use. However, these proportions are substantially smaller than those who reported ceased or decreased use of these substances (50.3% for illicit opioids, and 33.0% for cannabis). For all categories of non-psychedelic substances in the survey, the rates of increased or initiated use of these were highest for those living in the US or Canada. This is consistent with findings from a recent review on global substance use trends which showed that high income individuals in the North America region had among the most prevalent rates of dependence for cannabis, opioid, and cocaine use (53). Our regression results support this finding, as it was found that living in the US or Canada, and having a higher household income were both significantly associated with increasing or initiating use of other substances following naturalistic psychedelic use.

We remain cautious in drawing broad conclusions about the potential effectiveness of naturalistic psychedelic use for reducing substance use or treating SUD, and remain vigilant of possible adverse patient outcomes, such as increased or initiation of substance use. While it has been demonstrated that psychedelics generally have a lower potential for abuse compared to other substances, and the prevalence of substance use disorders associated with classic psychedelics is low (54, 55), naturalistic psychedelic use often occurs outside of clinical settings, may involve co-use with other substances that may be more habit forming, and is often not disclosed to relevant healthcare providers (34, 56). Therefore, additional work will be needed to better understand the relationships between naturalistic psychedelic use and substance use patterns to maximize the potential benefits of psychedelics while avoiding or reducing unnecessary harms.

### Implications

Although psychedelic-based treatments have shown increasing promise for the treatment of SUDs and other psychiatric conditions, the path toward implementation of these treatments into mainstream care will not be straightforward (57, 58). The results presented here provide evidence that psychedelic use may contribute to the alteration of substance use patterns, and therefore may be a potentially effective tool for addressing the large unmet need for treating SUDs. The current study, which benefits from a well-powered, global sample, suggests that naturalistic use—as opposed to clinical use—may also be an effective approach to changing substance use patterns for some individuals.

Among participants who used regular doses (not microdoses) of psilocybin, only 1.0% (51/5,052) reported doing so in a licensed clinic, wellness center, or hospital. This is an important finding for a few different reasons. First, mainstream acceptance and the expected widespread implementation of psychedelics into clinical practice suggests that the demand for psychedelic-based treatments is likely to exceed the supply of adequately trained health care professionals needed to oversee and provide support during psychedelic therapies. The current psychedelic-assisted therapy model requires one or more trained facilitators to be present during the multi-hour dosing sessions, in addition to providing preparatory and integration talk therapy. If psychedelic therapies are to meet the demands of millions of individuals with SUD and other mental health disorders, new approaches will be needed. And as more jurisdictions move toward the decriminalization or medicalization of psychedelic substances, it will be critical to continually monitor the effects of these policy changes on overall public health. The current findings suggest that, for a large majority of consumers, naturalistic psychedelic use is perceived to support overall reductions in the use of potentially more dangerous substances, which may supplement existing harm reduction strategies and contribute positive benefits to public health.

Second, the double-blind, placebo-controlled, randomized clinical trial model, which is typically referred to as the gold standard in psychotherapy and pharmacology research, is subject to several limitations that complicate the accurate discernment of treatment-specific effects of psychedelic therapies. These include small sample sizes, the lack of an effective placebo condition, inadequate masking of treatment conditions, as well as expectancy and selection biases among participants (59). The results presented here may shed light on a more ecologically valid view of global psychedelic use that mitigates some of the challenges associated with clinical research. However, we must not ignore the contribution that a regulated production/distribution model along with public education and medical oversight from trained professionals can provide to help ensure safety, recognize potential contraindications, and provide care in the case of adverse events. Indeed, the current study showed that a portion of the sample either initiated or increased use of another substance as a result of their psychedelic use, so it will be important to recognize this as a potential adverse effect of naturalistic psychedelic use. Future studies are needed to better understand long-term outcomes of naturalistic psychedelic use and investigate demographic, medical, and psychedelic use characteristics associated with differential outcomes, particularly as new regulations increase legal access to psychedelics for both medical and non-medical use.

### Limitations and strengths

The current study is limited in several ways. Because the survey relied on a cross-sectional assessment of self-reported substance use, we are unable to confirm the reported changes in psychedelic or non-psychedelic substance use patterns. Because of the reliance on self-report, we are also unable to confirm the extent to which perceived changes were correctly attributed to psychedelic use as a direct causal factor. Additionally, participant responses are subject to recall bias, and the convenience sampling strategy introduces a selection bias that prevents generalizability to a larger population of adults using psychedelics across the globe. The results are also limited because of recruitment biases, where recruitment strategies via primarily pro-psychedelic organizations may not have reached individuals who have had negative psychedelic experiences, or who do not follow such groups. Finally, the survey was only distributed in English, which resulted in an over-representation of White respondents and exclusion of non-English speaking individuals—an issue that we plan to address in future iterations.

Despite these limitations, this study is well-powered with a large sample size that spans dozens of countries across the world. This allows for an in-depth and comparative view of the relationships between naturalistic psychedelic use and the use of other licit and illicit non-psychedelic substances. We also collected extensive information on participant demographics and details about their psychedelic use that contributed to predictive regression modeling and a broader understanding of the factors associated with psychedelic use that may contribute to altered substance use patterns. As a result, these findings offer useful contributions that may inform future studies and policy considerations related to psychedelics.

### Conclusions

In this large, global survey of adults who self-reported using psychedelics naturalistically, 70.9% of the population reported ceasing or decreasing use of one or more non-psychedelic substances (e.g., alcohol, cannabis, tobacco/nicotine, antidepressants, amphetamines, cocaine/crack, prescription opioids, or illicit opioids) following naturalistic psychedelic use. Psilocybin was rated as the most impactful psychedelic leading to ceased or decreased use, and over a quarter of the population reported that their decrease in use lasted at least 26 weeks following psychedelic use. Logistic regression models showed that taking psychedelics with a motivation to either reduce one’s substance use, or to self-treat a medical condition were associated with decreased substance use. Explanatory factors associated with these changes related to increased connection to self, nature, spirit, and others, as well as altered perspectives on other substances. Nearly a quarter of participants reported increased use of one or more substances as a result of their psychedelic use, and predictive models indicated that having a higher income and living in Canada or the US were associated with those changes. These findings provide additional rationale for the need to investigate the potential of psychedelics for problematic substance use worldwide. Additionally, this large, observational study provides a unique approach to understanding psychedelic use, which mitigates some challenges associated with clinical investigation, and highlights the need for additional studies of naturalistic use. Future observational and clinical studies are warranted to develop a more nuanced understanding of the factors associated with altered substance use patterns, as well as to highlight additional considerations for safe and responsible psychedelic use.

## Data availability statement

The raw data supporting the conclusions of this article will be made available by the authors, without undue reservation.

## Ethics statement

The studies involving humans were approved by Advarra (protocol # Pro00071490) to help ensure that the rights and welfare of research participants were protected and that the research study was carried out in an ethical manner. However, the IRB only oversaw Canadian subjects, determining that this international study was otherwise exempt from IRB oversight in other jurisdictions under Department of Health and Human Services regulation 45 CFR 46.104(d)(2). The studies were conducted in accordance with the local legislation and institutional requirements. The participants provided their written informed consent to participate in this study.

## Author contributions

NG: Formal analysis, Investigation, Validation, Visualization, Writing – original draft, Writing – review & editing. JA: Writing – original draft, Writing – review & editing. DK: Methodology, Writing – review & editing. KB: Methodology, Supervision, Writing – original draft, Writing – review & editing. SL: Conceptualization, Investigation, Methodology, Writing – review & editing, Project administration. PL: Conceptualization, Funding acquisition, Investigation, Methodology, Supervision, Writing – review & editing, Project administration.

## Supplementary material

Supplementary Figure 1 Self-reported changes in substance use following psychedelic use among global regions. The number of participants who reported past or current use of each of the substances is listed below each substance. The results are separated based on current primary residence with A = Asia Pacific, M = Central Asia, Middle East, and Africa, E = Europe, L = Latin America, N = North America. Proportions for each category are listed in their respective locations, and values less than 5.0% are not shown.
