# Supplementary material for: Psychedelic substitution: altered substance use patterns following psychedelic use in a global survey
Source: Front Psychiatry. 2024 Feb 22;15:1349565. doi: 10.3389/fpsyt.2024.1349565 (PMC10917882; doi:10.3389/fpsyt.2024.1349565)
Supplement: Supplementary file 1 [file DataSheet_1.pdf]

SECTION HEADER: DEMOGRAPHICS

We would like to begin by asking you a few questions about yourself.

1. Which of the following best describes your gender?

- Male
- Female
- Non-binary
- Prefer not to say
- Prefer to self-describe:

2. How old are you?

[if Under 21 then end survey]

(DROP DOWN, Under 21-100)

3. Which of the following best describes your current relationship status?

- Single, never married
- Single, but cohabiting with a significant other
- In a domestic partnership or civil union
- Married
- Divorced / separated
- Widowed

4. Which of the following best describes your racial or ethnic background? (You may choose more than one)

- African (e.g., Central / West, South / East)
- Black / African diaspora (e.g., African-American, Caribbean)
- Caucasian / European
- East Asian (e.g., Chinese, Japanese, Korean)
- Indigenous (e.g., Native American, First Nations)
- Latin, Hispanic, Central and South American (e.g., Mexican, Colombian, Peruvian)
- Oceanian (e.g., Australia, New Zealand, surrounding islands)
- South Asian (e.g., Indian, Pakistani, Bangladeshi)
- South East Asian (e.g., Filipino, Indonesian, Malaysian)
- West Central Asian, Middle Eastern and North African (e.g., Armenian, Iranian, Lebanese, Egyptian, Moroccan)
- Prefer not to say
- Other, please specify:

5. In which country do you reside?

POPULATE WITH DROP DOWN OF WORLD COUNTRIES

6. In which country were you born?

POPULATE WITH DROP DOWN OF WORLD COUNTRIES

7. Do you currently live in a:

- Rural or remote area
- Suburban area
- Urban area

8. What is the highest level of school you have completed or the highest degree you have received?

- Less than high school degree
- High school degree or equivalent
- Technical and non-university degree (college; CEGEP)
- University degree (Bachelors' degree or equivalent)
- Graduate degree (MA, MSc, etc.)
- Doctorate or professional degree (JD, MD, PhD, etc.)

9. Which of the following categories best describes your employment status?

- Employed, working full-time
- Employed, working part-time
- Not employed, looking for work
- Not employed, NOT looking for work
- Retired
- Disabled, not able to work

10. Would you consider your household income (the total for you and your spouse / partner, if applicable) to be:

- Very low income / well below average
- Low income / below average
- Middle income / about average
- High income / above average
- Very high income / well above average

===

SECTION HEADER: SUBSTANCE USE

We'd like to start by asking you about your use of psychoactive substances OTHER than psychedelics.

11. **IN YOUR LIFETIME**, have you ever used any of the following substances? **(YES/NO BOXES FOR EACH ON GRID)**

a. Alcohol
- Yes
- No

b. Amphetamines / methamphetamines, as prescribed (e.g., Ritalin, Adderall)
- Yes
- No

c. Amphetamines / methamphetamines, non-prescribed / recreational (e.g., speed, crystal meth)
- Yes
- No

d. Benzodiazepines, as prescribed (e.g., Valium, Ativan, etc.)
- Yes
- No

e. Benzodiazepines, non-prescribed / recreational (Valium, Ativan, etc.)
- Yes
- No

f. Cannabis, as prescribed
- Yes
- No

g. Cannabis, non-prescribed / recreational
- Yes
- No

h. Cocaine / crack
- Yes
- No

i. Opioids, as prescribed (e.g., fentanyl, oxycodone, hydromorphone, etc.)
- Yes
- No

j. Opioids, non-prescribed/recreational (e.g., heroin, fentanyl, oxycodone, hydromorphone, etc.)
- Yes
- No

k. Tobacco / nicotine
- Yes
- No

IF "YES" TO ANY IN PREVIOUS QUESTION, USE PIPING TO POPULATE ONLY WITH SUBSTANCES IDENTIFIED AS BEING USED OVER LIFETIME (IF POSSIBLE)

12. **IN THE PAST 12 MONTHS**, have you used any of the following substances? **(YES/NO BOXES FOR EACH ON GRID)**

If answer to Question 11 = "Yes", then it will be shown again here with the options of:

- Yes
- No

===

SECTION HEADER: PSYCHEDELIC USE

We'd now like to ask you a few questions about your use of psychedelics.

13. How old were you when you first tried psychedelic substances (e.g., ketamine, psilocybin, MDMA, LSD, etc.)?

DROP DOWN, 1-100

14. Please rank your current level of **KNOWLEDGE** of psychedelic substances from 0 - 100, with 0 being "No knowledge at all" and 100 being "Very knowledgeable".

INSERT SLIDING SCALE, 0-100
(Place a mark on the scale above)

15. Please rank your current level of **EXPERIENCE** with psychedelic substances from 0-100, with 0 being "No experience at all" and 100 being "Very experienced".

INSERT SLIDING SCALE, 0-100
(Place a mark on the scale above)

16. What are the reasons you use psychedelic substances? Check all that apply.

- ❖ To treat a medical condition (physical or mental health)
- ❖ To reduce my use of another substance (prescription or non-prescription)
- ❖ General well-being (i.e., to improve overall mental or physical functioning but not to treat a specific disease or illness or symptoms caused by disease / illness)
- ❖ Personal growth / self-exploration

- ❖ Religious / spiritual development
- ❖ Recreational (e.g., for fun, social connectedness, euphoria)
- ❖ None of the above

17. Have you taken a psychedelic substance in the **PAST YEAR / 12 MONTHS**?

- Yes
- No

IF YES TO Q17:

17a. Approximately how frequently have you taken psychedelic substances in the **PAST YEAR / 12 MONTHS**?

- Once
- Once every 6 months
- Once every 2-5 months
- Once every month
- Once every week
- 2-3 times per week
- Daily

17b. How have you purchased or accessed psychedelic substances in the **PAST YEAR / 12 MONTHS**? Check all that apply.

- I received them from a friend / acquaintance
- I bought them from a dealer (in-person or online)
- I bought them through an online store / retailer
- I bought them in-person at a retail dispensary
- I accessed them via a clinic and / or a healthcare professional (e.g., regulated ketamine clinic)
- I bought or received them from a pharmacy
- I accessed them via an underground therapist / practitioner
- I accessed them via shaman, curandero or spiritual leader
- I produced and / or harvested them myself
- None of the above

17c. Ideally, what would be your preferred way(s) to purchase and / or access psychedelic substances? Check all that apply.

- From a friend / acquaintance
- From a dealer (in-person or online)
- Through an online store / retailer

- In-person, at a retail dispensary
- From a clinic and / or a healthcare professional (e.g., regulated ketamine clinic)
- From a pharmacy
- From a shaman, curandero or spiritual leader
- I would produce / harvest them myself
- None of the above

===

SECTION HEADER: PATTERNS OF USE

18. **IN YOUR LIFETIME**, have you ever used any of the following psychedelic substances?

[If no to all substances then end survey]

2C-B
- Yes
- No

Ayahuasca
- Yes
- No

DMT / 5-MeO-DMT
- Yes
- No

Iboga / Ibogaine
- Yes
- No

Ketamine (K)
- Yes
- No

LSD / Acid
- Yes
- No

MDMA / MDA (Ecstasy / Molly)
- Yes
- No

Mescaline (San Pedro, Peyote, etc.)
- Yes
- No

Nitrous Oxide (non-dental, e.g., whippits)
- Yes
- No

Psilocybin (mushrooms or synthetic)

- Yes
- No
- Yes

Salvia Divinorum

- Yes
- No

Other psychedelic drug, please specify

- Yes
- No

IF YES TO Q18:

18a. **IN YOUR LIFETIME**, approximately how many times have you used the following substances?

If answer to Question 18 = "Yes", then it will be shown again here with the options of:

- numeric input (1-1000)

18h. Where do you TYPICALLY obtain the following psychedelic substances?

If the answer to Question 18 = "Yes", then it will be shown again here. Answer options for each are the following:

- I receive them from a friend / acquaintance
- I buy them from a dealer (in-person or online)
- I buy them through an online store / retailer
- I buy them in-person at a retail dispensary
- I access them via a clinic and / or a healthcare professional (e.g., regulated ketamine clinic)
- I buy or receive them from a pharmacy
- I access them via an underground therapist/practitioner
- I access them via shaman, curandero or spiritual leader
- I produce and / or harvest them myself
- None of the above

The next series of questions will ask you about both REGULAR DOSING and MICRODOSING of psychedelic substances. We define a REGULAR DOSE "regular dose" as leading to psychedelic effects (i.e., a "trip"), while a MICRODOSE is typically a small fraction of a "regular dose" and too low to produce a "trip".

18b. **IN YOUR LIFETIME**, have you ever used the following substances in regular doses, microdoses or both?

If answer to Question 18 = "Yes", then it will be shown again here with the options of:

- Regular doses ONLY (no microdoses)
- Microdoses ONLY (no regular doses)
- BOTH regular doses AND microdoses

IF 18C = "REGULAR DOSES ONLY (NO MICRODOSES)" OR "BOTH REGULAR DOSES AND MICRODOSES"

18d. Have you used **REGULAR DOSES** of following substances over the **PAST 12 MONTHS**?

If the answer to Question 18 = "Yes", then it will be shown again here. If an "Other" substance was specified, the entered text will appear here. Answer options for each are the following:

- Yes
- No

[GPS-REGULAR-DOSE-INTRO]

Now we'd like to ask you some questions related to your TYPICAL patterns of use with REGULAR DOSES of specific psychedelic substances (NOT microdoses). TYPICAL refers to your most common / average experience with each substance. If you've only used a REGULAR DOSE of the substance once, please refer to that experience. If you indicated microdosing any psychedelic substances, we will ask you about typical experiences with microdosing later.

18c. How often do you **TYPICALLY** use a **REGULAR DOSE** of the following substances?

If the answer to Question 18 = "Yes", then it will be shown again here. Answer options for each are the following:

- Less than once per year
- Once or twice per year
- Less than once per month
- 1 or 2 times per month
- Once per week

- 2-3 times per week
- Every 2 days
- Every day

18e. With whom do you **TYPICALLY** use **REGULAR DOSES** of the following psychedelic substances?

If the answer to Question 18 = "Yes", then it will be shown again here. Answer options for each are the following:

- No one (I typically use on my own)
- A trip-sitter (an abstaining companion who monitors your safety)
- A licensed therapist or health professional
- An unlicensed therapist, shaman, trip guide, and/or spiritual group
- A spouse, companion or a few friends
- A larger social group (e.g., friends, acquaintances, and/or strangers)
- None of the above

18f. In which setting do you **TYPICALLY** use **REGULAR DOSES** of the following psychedelic substances?

If the answer to Question 18 = "Yes", then it will be shown again here. Answer options for each are the following:

- Indoors in a familiar setting (e.g., my home or a friend's house)
- In a licensed clinic, wellness centre or hospital
- At an underground therapist / practitioner's office or clinic
- At a small gathering / retreat
- At a large public gathering / party / rave / event
- Outdoors in a natural environment (e.g., in a park or forest)
- None of the above

18g. **TYPICALLY**, what is the primary / main intent of your use of **REGULAR DOSES** of the following substances?

If the answer to Question 18 = "Yes", then it will be shown again here. Answer options for each are the following:

- To treat a medical condition (physical or mental health)
- To reduce my use of another substance (prescription or non-prescription)

- To increase general well-being (i.e., to improve overall mental or physical functioning but not to treat a specific disease or illness or symptoms caused by disease / illness)
- For personal growth / self-exploration
- For religious / spiritual development
- For recreational purposes (e.g., for fun, social connectedness, euphoria)
- None of the above

18i. When you use REGULAR DOSES of the following psychedelics, do you TYPICALLY use other substances such as alcohol, benzos, tobacco, cannabis or other drugs/psychedelics (i.e., 1-2 hours before, around the same time as, or 1-2 hours after)?

If the answer to Question 18 = "Yes", then it will be shown again here. Answer options for each are the following:

- Yes
- No

If YES:

MATRIX

18j. Do you typically consume any of the following substances 1-2 hours before, during, or 1-2 hours after you use **REGULAR DOSES** of the following substances? (Please click all that apply.)

If the answer to Question 18 = "Yes", then it will be shown again here. If Question 11 = "Yes" then it will appear here as an answer option for the above.

The populated list of answer options should NOT contain the substance in question. (i.e. you cannot mix the same substance with itself)

Answer options for each are the following:

- 1 - 2 hours before
- During
- 1 - 2 hours after

If ANY yes response to Question 18i is yes.

18k. TYPICALLY, what is your intent when using another substance 1-2 hours **BEFORE** a psychedelic substance?

- To increase / enhance the psychedelic experience
- To decrease / reduce the psychedelic experience
- None of the above
- N/A - I don't typically use another substance 1-2 hours before using any psychedelic substances

If ANY response to Question 18i is yes.

18l. TYPICALLY, what is your intent when using another substance **AROUND THE SAME TIME** as a psychedelic substance?

- To increase / enhance the psychedelic experience
- To decrease / reduce the psychedelic experience
- None of the above
- N/A - I don't typically use another substance around the same time as any psychedelic substances

If ANY response to Question 18i is yes

18m. TYPICALLY, what is your intent when using another substance 1-2 hours **AFTER** a psychedelic substance?

- To increase / enhance the psychedelic experience
- To decrease / reduce the psychedelic experience
- None of the above
- N/A - I don't typically use another substance 1-2 hours after using any psychedelic substances

IF 18C = "MICRODOSES DOSES ONLY" OR "BOTH REGULAR DOSES AND MICRODOSES"

19. Have you **MICRODOSED** the following psychedelic substances over the **PAST 12 MONTHS**?

If the answer to Question 18b includes microdosing , then it will be shown again here. Answer options for each are the following:

- Yes
- No

Now we'd like to ask you some questions related to your **TYPICAL** patterns of use with **MICRODOSES** of specific psychedelic substances. **TYPICAL** refers to your most common / average experience with each substance. If you've only used a **MICRODOSE** of the substance once, please refer to that experience.

20a. How often do you typically **MICRODOSE** the following substances?

Options selected in Question 18b.

- Less than once per year
- Once or twice per year
- Less than once per month
- 1 or 2 times per month
- Once per week
- 2-3 times per week
- Every 2 days
- Every day

19a. TYPICALLY, what is your primary / main intent for **MICRODOSING** the following psychedelic substances?

If the answer to Question 18b includes microdosing, then it will be shown again here. Answer options for each are the following:

- To treat a medical condition (physical or mental health).
- To reduce my use of another substance (prescription or non-prescription)
- For general well-being (i.e., to improve overall mental or physical functioning but not to treat a specific disease or illness or symptoms caused by disease / illness).
- For personal growth / self-exploration.
- Religious / spiritual development.
- To increase productivity / motivation/creativity
- To increase energy / stimulation
- To increase mindfulness
- To enhance the effects of another substance
- To experience a pleasurable effect without "tripping"
- None of the above

[AFTER ALL PSYCHEDELIC SUBSTANCE-SPECIFIC REGULAR- AND MICRODOSE QUESTIONS ASKED ABOUT 11 PRIMARY PSYCHEDELICS]: IF SELECTED "MICRODOSE(S)" TO *ANY* SUBSTANCE:

SECTION HEADER: PSYCHEDELICS AND MUSIC

We would now like to ask you a few questions about psychedelics and music.

21. Have you ever experienced psychedelics **WITH** music?

- Never
- 1-5 times
- 6-10 times
- >10 times

For Q22-27, IF ANYTHING EXCEPT "NEVER" in Q21

22. Have you experienced psychedelics **WITHOUT** music?

- Never
- 1-5 times
- 6-10 times
- >10 times

23. When choosing music for psychedelic experiences do you:

- Create your own music playlist
- Use existing playlists created by others
- Just play some music in the moment without planning
- Listen to live music played by others
- Play musical instruments by myself
- Play musical instruments with others
- None of the above

24. What music genres do you find particularly suited for psychedelic experiences? Check all that apply.

- Classical
- Electronic dance music
- Ambient / Electronica
- World music
- Pop music
- Rock
- Jam bands (Grateful Dead, Phish, etc.)
- Jazz
- Country / Blues
- Hip-hop / Rap
- Meditative / New Age
- Music from religious / spiritual traditions
- Other, please specify:

25. Please indicate your level of agreement with the following statements about music:
Answer options for each: Strongly agree, Somewhat agree, Neither agree nor disagree, Somewhat disagree, Strongly disagree

a. Generally, music is very important to me.
b. Generally, I often listen to music.
c. Generally, I only enjoy specific genres of music.
d. Generally, in group settings, I often select the music.
e. For psychedelic experiences, music should be unfamiliar.
f. For psychedelic experiences, music should be without understandable lyrics.
g. To maximize their **BENEFITS** psychedelic experiences should include periods **WITH** music.
h. To maximize **ENJOYMENT** psychedelic experiences should include periods **WITH** music.
i. To maximize their **BENEFITS** psychedelic experiences should include periods **WITHOUT** music.
j. To maximize **ENJOYMENT** psychedelic.experiences should include periods **WITHOUT** music.
k. Psychedelic experiences have changed my relationship with music.

Q26, IF "AGREE" OR "SOMEWHAT AGREE" TO THE LAST QUESTION IN THE MATRIX ABOVE (25k):

26. How have psychedelic experiences changed your relationship with music? Check all that apply.

- Increased the importance of music in my life
- Decreased the importance of music in my life
- Increased my enjoyment of music in general
- Decreased my enjoyment of music in general
- Increased my enjoyment of specific genres of music
- Decreased my enjoyment of specific genres of music
- Increased my desire to create music
- Decreased my desire to create music

27. Are there any of the following psychedelics that you like or don't like to experience with music?

If the answer to Question 18 = "Yes", then it will be shown again here. Answer options for each are the following:

- Prefer with music
- Prefer with combination of music / no music

- Prefer with no music
- No preference
- Never used this substance with music

SECTION HEADER: VISUAL HALLUCINATIONS

We'd now like to ask you about different types of vivid visual hallucinations you may have experienced while taking psychedelic substances.

28. During a psychedelic experience, have you experienced **VIVID VISUAL HALLUCINATIONS** resembling media that you have recently seen (e.g., television shows, video games, paintings, etc.)?

- Never
- Occasionally
- About half the time
- Frequently
- Always

29. During a psychedelic experience, have you experienced **VIVID VISUAL HALLUCINATIONS** resembling things that you have recently seen other than media (e.g., particular landscapes, cities, etc.)?

- Never
- Occasionally
- About half the time
- Frequently
- Always

IF ANYTHING BUT "NEVER" TO 28 OR 29:

30.Do these **VIVID VISUAL HALLUCINATIONS** occur more often when using a specific psychedelic substance?

- yes
- no

IF YES TO 30, then please populate with list of all psychedelics tried in lifetime from Q18

30a. Please check off any of the following psychedelic substances which result in an increase of **VIVID VISUAL HALLUCINATIONS** of media or things you have seen recently other than media (Please check all that apply).

- *If the answer to Question 18 = "Yes", then it will be shown again here.*

- *None of the above (exclusive)*

SECTION HEADER: LONGER TERM HALLUCINATIONS

We'd now like to ask you about potential hallucinations you may have experienced MORE THAN 24 HOURS AFTER the psychedelic drug effect has subsided.

31. Have you ever experienced lasting hallucinations **MORE THAN 24 HOURS AFTER** the psychedelic drug effect has subsided?

- Never
- Occasionally
- About half the time
- Frequently
- Always

IF ANYTHING EXCEPT "NEVER" show questions 32-34a

32. How do these lasting hallucinations **MORE THAN 24 HOURS AFTER** the psychedelic drug effect has subsided impact your life?

- Very negative impact
- Somewhat negative impact
- No impact
- Somewhat positive impact
- Very positive impact

33. In number of days, how long do these hallucinations that take place **MORE THAN 24 HOURS AFTER** the psychedelic drug has subsided typically last?

DROP DOWN OF 1-100, WITH ADDITION OF "OVER 100 DAYS" AT THE END

---

34. Do these hallucinations that take place **MORE THAN 24 HOURS AFTER** the psychedelic drug has subsided occur more often when using a specific psychedelic substance?

- yes
- no

IF YES TO 34, then please populate with list of all psychedelics tried in lifetime from Q18

34a. Please check off any of the following psychedelic substances which result in hallucinations that take place **MORE THAN 24 HOURS AFTER** the psychedelic drug has subsided (Please check all that apply).

- *If the answer to Question 18 = "Yes", then it will be shown again here.*
- *None of the above*

SECTION HEADER: NATURAL VS SYNTHETIC

We'd now like to ask you a few questions about the use of natural and synthetic sources of psychedelics.

35. Given the option, would you prefer to use a natural or synthetic, lab-derived versions of the following psychedelics:

Answer options: Natural, Synthetic, No preference, N/A - I have no interest in ever using this substance

a. 5-MeO-DMT (natural source: bufo toad)
b. DMT (natural source: ayahuasca vine, chacruna, etc.)
c. Iboga / Ibogaine (natural source: iboga root)
d. Mescaline (natural source: San Pedro, Peyote, etc.)
e. Psilocybin / psilocin (natural source: mushrooms)

36. How strongly do you agree or disagree with the following statement:
Despite being chemically identical, the source (i.e., natural vs. synthetic/lab-derived) of a psychedelic substance can impact its psychological/physiological effects.

- Strongly disagree
- Somewhat disagree
- Neutral / undecided
- Somewhat agree
- Strongly agree

37. How strongly do you agree or disagree with the following statement:
If it would lessen our impacts on the environment and on plant and animal species like the Bufo toad (5-MeO-DMT), peyote cactus (mescaline), iboga plant (iboga) and ayahuasca vine (DMT), I would consider using synthetic/lab-derived alternatives instead of naturally-sourced versions of these substances if they were available.

- Strongly disagree
- Somewhat disagree
- Neutral / undecided
- Somewhat agree
- Strongly agree

SECTION HEADER: QUALITY AND POTENCY TESTING

We'd now like to ask you a few questions about quality and potency testing of psychedelics.

38. Are you aware of the availability of laboratory services or at home test kits to confirm the identity of psychedelic substances?

- Yes
- No

IF YES to Q36:

38a. How often do you use laboratory services and/or at home test kits to confirm the identity of a psychedelic substance before consuming?

- Never
- Rarely
- Sometimes
- Often
- Always

IF ANYTHING EXCEPT "NEVER" IN Q38a:

39. What kind of testing service do you use? Check all that apply.

- At home test kit
- Testing service at event (e.g., on-site at a music festival)
- My dealer, therapist or shaman tests psychedelics before use
- Lab-based testing service
- Other, please describe:

SECTION HEADER: INTENSITY OF EXPERIENCE (adapted from Awe Experience Scale (AWE-S))

We'd now like to ask you a few questions related to the intensity of psychedelic experiences and associated outcomes. An "intense" psychedelic experience may mean encountering and processing complex positive emotions and thoughts, negative or challenging emotions and thoughts, or a mix of both, while using a psychedelic substance.

[LSL1]We have changed this section slightly. The goal of this section is to ask about the person's most intense psychedelic experience – whether positive, challenging/negative, or both – and to document details about that experience.

We have changed the wording of some of the follow-up questions about the person's "most intense positive" and "most intense negative/challenging" experience so that those questions are still gathering follow-up data on the positive/negative aspects of the person's *most intense* experience (rather than 2 separate experiences – 1 positive, 1 negative – as was implied in the earlier version).

40. Have you ever had what you would describe as an **INTENSE** experience while using psychedelics?

- Yes
- No

IF YES TO Q40:

45. What psychedelic substance were you using when you had your **MOST INTENSE PSYCHEDELIC EXPERIENCE**?

- *If the answer to Question 18 = "Yes", then it will be shown again here.*

- **None of the above**

41. Please indicate your level of agreement with the following statements about your MOST INTENSE EXPERIENCE with psychedelics.
Answer options: Strongly disagree (1), Moderately disagree (2), Somewhat disagree (3), Neutral (4), Somewhat agree (5), Moderately agree (6), Strongly agree (7)

a. I sensed things momentarily slow down.
b. I noticed time slowing.

c. I felt my sense of self was diminished.
d. I felt my sense of self shrink.

e. I had the sense of being connected to everything.
f. I felt a sense of communion with all living things.

g. I felt that I was in the presence of something grand.

h. I experienced something greater than myself.

i. I felt my jaw drop.
j. I had goosebumps.

k. I felt challenged to mentally process what I was experiencing.
l. I found it hard to comprehend the experience in full.

42. At the time of your **MOST INTENSE EXPERIENCE**, did you consider it to be:

- Positive / pleasurable / desirable
- Negative / challenging / detrimental
- A mix of both positive / pleasurable / desirable AND negative / challenging / detrimental

43. Do you believe that your **MOST INTENSE EXPERIENCE** and your contemplation of that experience led to a **POSITIVE or NEGATIVE** change in your **CURRENT** sense of personal well-being or life satisfaction?

- Strong positive change that I consider desirable
- Moderate positive change that I consider desirable
- Slight positive change that I consider desirable
- No change
- Slight negative change that I consider detrimental
- Moderate negative change that I consider detrimental
- Strong negative change that I consider detrimental

IF ANYTHING BUT "NO CHANGE" in Q43:

43a. If changed, please describe the specific changes:

____

46. On a scale of 0 to 100, with 0 being "worst experience of my life" (equal to the death of a loved one or other traumatic loss, etc.) and 100 being "best experience of my life" (equal to marriage, birth of a child, etc.), how would you rank your **MOST INTENSE psychedelic experience**?

INSERT SLIDING SCALE, 0-100

0. Worst experience	50. Neutral	100. Best experience

(Place a mark on the scale above)

53. Did you experience any of the following during your **MOST INTENSE psychedelic experience**? Check all that apply.

- I had to confront challenging personal life issues (e.g., loss, past physical or psychological trauma, addiction, etc.)
- I felt social paranoia (felt as if the world was hostile or malicious)
- I experienced troubling or menacing visions
- I experienced mental or sensory overload
- I experienced ego death / dissolution of the self
- I experienced physical, emotional or sexual abuse
- I worried about my mental or physical health
- I worried that I would never be the same after the experience
- I worried that I might be arrested
- I worried that I might be assaulted
- I worried that I might be hospitalized
- I worried that I might die
- None of the above

47. Who were you with during your **MOST INTENSE psychedelic experience**?

- No one (I was alone)
- A trip-sitter (an abstaining companion who monitors your safety)
- A licensed therapist or health professional
- An unlicensed therapist, shaman, trip guide, and/or spiritual group
- A spouse, companion or a few friends
- A larger social group (e.g., friends, acquaintances, and/or strangers)
- None of the above

48. What was the setting of your **MOST INTENSE psychedelic experience**?

- Indoors in a familiar setting (e.g., my home or a friend's house)
- In a licensed clinic, wellness centre or hospital
- At an underground therapist / practitioner's office or clinic
- At a small gathering / retreat
- At a large public gathering / party / rave / event
- Outdoors in a natural environment (e.g., in a park or forest)
- None of the above

IF NEGATIVE or MIX OF POSITIVE AND NEGATIVE TO Q42:

54. Looking back on your **MOST INTENSE psychedelic experience**, which of the following statements best characterizes the outcome of that experience?

- No good came from this challenging experience; it was all bad
- Very little good came from this challenging experience; it was mostly bad
- A balance of good and bad came from this challenging experience
- Some significant good came from this challenging experience; the good largely outweighed the bad
- A great deal of good came from this challenging experience; the good strongly outweighed the bad

IF ANYTHING EXCEPT “NO GOOD” or “VERY LITTLE GOOD” to Q54:

55. What specific good came from this **INTENSE psychedelic experience**? Check all that apply.

- Resolved a challenging situation or emotion
- Gained insight into a very difficult problem or life issue
- Overcame some of my personal fears
- Addressed long-standing physical or psychological trauma(s)
- Experienced "ego death" (a dissolution of the self, and feeling of oneness with the world / universe)
- Other, please specify:

SECTION HEADER: PSYCHEDELICS AND HEALTH

We’d now like to ask you a few questions about psychedelics and your psychological and physical health.

56. Do you regularly see a primary healthcare provider (i.e. medical doctor, general practitioner, community healer, etc.)?

- Yes
- No

IF NO to Q56 skip to Q60

57. Have you ever discussed psychedelics with your primary healthcare provider?

- Yes
- No

IF NO to Q57:

57a. Why haven't you discussed psychedelics with your primary healthcare provider? Check all that apply.

- Concerns about stigma associated with psychedelic use
- I prefer to keep my psychedelic use private
- Legal concerns
- To avoid contradicting my healthcare provider's advice
- I do not trust my primary healthcare provider
- My primary healthcare provider is not adequately knowledgeable about psychedelics
- I do not believe my primary healthcare provider would be able to integrate psychedelic use into my treatment
- I do not feel there's any reason to discuss my psychedelic use with my healthcare provider
- None of the above

58. How comfortable are you sharing details about the consequences of your psychedelic use (such as changes in symptoms or changes in use of medication) with your primary healthcare provider?

- Very comfortable
- Somewhat comfortable
- Neither comfortable nor uncomfortable
- Somewhat uncomfortable
- Very uncomfortable

59. How would you rate your primary healthcare provider's knowledge of psychedelics?

- Excellent
- Very good
- Good
- Fair
- Poor
- I don't know

60. Have you used psychedelics to treat a **PHYSICAL OR MENTAL HEALTH** symptom or condition? Check all that apply.

- Yes, I have used psychedelics to treat a PHYSICAL HEALTH condition
- Yes, I have used psychedelics to treat a MENTAL HEALTH condition
- No, I have never used psychedelics to treat a physical or mental health condition

IF either YES TO Q60 (if no, go straight to #79:

60a. What led to you trying psychedelics for a **PHYSICAL OR MENTAL HEALTH** symptom or condition? Check all that apply.

- Traditional treatments had failed or been ineffective
- I didn't like the idea of traditional treatments
- It fits in with my beliefs or cultural practices
- It was recommended by friends
- I saw positive media coverage
- Other (describe)
- None of the above

IF YES TO PHYSICAL HEALTH IN Q60:

61. Which **PHYSICAL HEALTH** condition or symptom have you treated with psychedelics? Check all that apply.

- Autism spectrum disorder
- Cancer
- Cardiovascular condition (e.g., heart disease)
- Chronic pain (e.g., fibromyalgia)
- Covid-19
- Diabetes
- Gastrointestinal disorder (e.g., Crohn's disease, colitis)
- Headache / migraines
- Neurological disorder (e.g., epilepsy)
- Sleeping disorder (e.g., insomnia)
- Traumatic Brain Injury / head trauma
- Other, please specify:

62a. Have you found the use of psychedelic substances to be effective in treating your **PHYSICAL HEALTH** condition(s)?

- Yes
- No

[If Yes to 62a]:
62b. Which psychedelic has been **MOST EFFECTIVE** in treating your **PHYSICAL HEALTH** condition(s)?

- *If the answer to Question 18 = "Yes", then it will be shown again here.*

- *None of the above*

63. How effective is the following psychedelic substance in relieving your PHYSICAL HEALTH symptoms / conditions?

INSERT SLIDING SCALE, 0-100
0% - not at all effective 50% - somewhat effective 100% - very effective
(Place a mark on the scale above)

IF ANYTHING BUT “0” in Q63

64. How long do the benefits of psychedelic use on your **PHYSICAL HEALTH** condition typically last?

- Less than 1 week
- 1 week
- Several weeks but less than a month
- 1 month
- Several months but less than a year
- A year or two
- More than two years

IF YES TO MENTAL HEALTH in Q60:

65. Which **MENTAL HEALTH** condition or symptom have you treated with psychedelics? Check all that apply.

- Addiction / withdrawal / substance use disorder
- Anxiety
- Attention Deficit Hyperactivity Disorder (ADHD)
- Bipolar disorder
- Depression / low mood
- Eating disorder (e.g., anorexia)
- Obsessive Compulsive Disorder (OCD)
- Personality disorder (e.g., Borderline Personality Disorder)
- Post-Traumatic Stress Disorder (PTSD)
- Psychotic disorder (e.g., schizophrenia)
- Other, please specify:

66a) Have you found the use of psychedelic substances to be effective in treating your MENTAL HEALTH condition(s)?

- Yes
- No

[If No, proceed to 70]

66b). Which psychedelic has been **MOST EFFECTIVE** in treating your **MENTAL HEALTH** condition(s)?

- *If the answer to Question 18 = "Yes", then it will be shown again here. Answer options for each are the following:*

SLIDING SCALE:
67. How effective is the following substance in relieving your **MENTAL HEALTH** symptoms/conditions?

INSERT SLIDING SCALE, 0-100

0% - not at all effective 50% - somewhat effective 100% - very effective

(Place a mark on the scale above)

IF ANYTHING BUT “0” in Q67

68. How long do the benefits from psychedelic use on your **MENTAL HEALTH** condition typically last?

- Less than 1 week
- 1 week
- Several weeks but less than a month
- 1 month
- Several months but less than a year
- A year or two
- More than two years

70. Prior to using psychedelics for **MENTAL HEALTH** did you pursue any of these conventional treatment options? Check all that apply.

- Talk therapy / counselling
- Cognitive-behavioural therapy

- Mindfulness
- Medications (e.g., antidepressants, antianxiety, mood stabilizers, antipsychotics, sedatives)
- Combination therapy and medications
- Herbal remedies (e.g., St. John's Wort, Kava Kava, Chamomile Extract, Valeria)
- Meditation / yoga / Tai Chi / Qigong
- Physical activity
- No, I did not pursue any of these traditional treatment options

71. Do you **CURRENTLY** pursue any of the following traditional treatment options for your **MENTAL HEALTH**? Check all that apply.

- Talk therapy/counselling
- Cognitive-behavioural therapy
- Mindfulness
- Medications (e.g., antidepressants, antianxiety, mood stabilizers, antipsychotics, sedatives)
- Combination therapy and medications
- Herbal remedies (i.e. St. John's Wort, Kava Kava, Chamomile Extract, Valeria)
- Meditation / yoga / Tai Chi / Qigong
- Physical activity
- No, I do not currently pursue any of these traditional treatment options

72. From what type of practitioner have you received **MENTAL HEALTH** treatment? Check all that apply.

- A mental health counsellor
- A psychotherapist
- A family doctor / general practitioner
- A psychologist
- A social worker
- A psychiatrist
- A religious / spiritual healer
- Other (please specify)
- I have not seen a mental health practitioner

IF YES TO MENTAL HEALTH COUNSELLOR, PSYCHOTHERAPIST, PSYCHOLOGIST, OR PSYCHIATRIST in Q72

73. Have you let your mental health counsellor, psychotherapist, psychologist, psychiatrist or therapist know you are using psychedelics?

- Yes
- No

IF NO TO Q73 show Q74

74. What are the reasons that you have not disclosed this information to mental health counsellor, psychotherapist, psychologist, psychiatrist or therapist? Check all that apply.

- Concerns about stigma associated with psychedelic use
- I prefer to keep my psychedelic use private
- Legal concerns
- To avoid contradicting my therapist's advice
- I do not trust my therapist
- My therapist is not adequately knowledgeable about psychedelics
- I do not believe my therapist would be able to integrate psychedelic use into my treatment
- I don't feel there's any reason to discuss my psychedelic use with my therapist
- None of the above

75. Have you ever used psychedelics under the care and guidance of a therapist or health care professional?

- Yes
- No

IF YES TO Q75:
76. How important was the presence of the therapist or health care professional to the overall outcome of your psychedelic experience?

- Very important
- Important
- Moderately important
- A little important
- Not important

IF NO TO Q75

77. How likely is it that you would consume psychedelics under the guidance or support of a trained therapist or health care professional if these services were legal and available to you?

- Very likely
- Likely
- Neutral
- Unlikely
- Very unlikely

IF (YES TO Q66A) AND (“Talk therapy / counselling” OR “Cognitive behavioural therapy” OR “Medications (e.g., antidepressants, antianxiety, mood stabilizers, antipsychotics, sedatives)” OR “Combination therapy and medications” to Q70):

78. Have you reduced or discontinued your use of any of the following traditional treatments since using psychedelics to treat your **MENTAL HEALTH** condition? Check all that apply

- Present each eligible option from Q70 that they ticked off
- No

.

79. GAD-7. Over the **LAST 2 WEEKS**, how often have you been bothered by the following problems?
Answer options: Not at all, Several days, More than half the days, Nearly every day

a. Feeling nervous, anxious or on edge
b. Not being able to stop or control worrying
c. Worrying too much about different things
d. Trouble relaxing
e. Being so restless that it is hard to sit still
f. Becoming so easily annoyed or irritable
g. Feeling afraid as if something awful might happen

80. PHQ-8: **OVER THE PAST 2 WEEKS**, how often have you been bothered by any of the following problems?
Answer options: Not at all, Several days, More than half the days, Nearly every day

a. Little interest or pleasure in doing things
b. Feeling down, depressed or hopeless
c. Trouble falling/staying asleep or sleeping too much
d. Feeling tired or having little energy
e. Poor appetite or overeating
f. Feeling bad about yourself, or that you are a failure or have let yourself or your family down
g. Trouble concentrating on things, such as reading the newspaper or watching television
h. Moving or speaking so slowly that other people could have noticed. Or the opposite, being so fidgety or restless that you have been moving around a lot more than usual.

SECTION HEADER: HEADACHES

ONLY FOR THOSE THAT IDENTIFY HEADACHE / MIGRAINES AS A REASON WHY THEY USE PSYCHEDELICS FOR THERAPEUTIC PURPOSES (Q61)

We'd now like to ask you a few questions about your use of psychedelics to treat headaches and migraines.

81. Do you have a medical diagnosis of what type of headache you are treating with psychedelics?

- Migraine
- Cluster headache
- Other (manually enter headache diagnosis being treated)
- I don't have a medical diagnosis for my headaches

82. Have your headaches interfered with your ability to work, study, or do what you needed to do?

- Yes
- No

83. Have you felt nauseated or sick to your stomach when you have a headache?

- Yes
- No

84. Does light bother you when you have a headache (a lot more than when you don't have a headache)?

- Yes
- No

85. Which of the following psychedelics have you used to treat headaches / migraines? Check all that apply.

- *If the answer to Question 18 = "Yes", then it will be shown again here.*
- *None of the above*

86. Which of the following has been most effective in treating your headaches / migraines?

- *If the answer to Question 18 = "Yes", then it will be shown again here. If an "Other" substance was specified, the entered text will appear here.*
- None - no psychedelic has been effective in treating my headaches / migraines

87. Do you use psychedelics as an abortive (as needed) treatment for your headaches, or as a preventive treatment?

- Abortive
- Preventative
- Both abortive and preventative treatment

88. Do you use "regular" dosing or are you "microdosing" to treat headaches / migraines?

- Regular dosing
- Microdosing
- Both regular and microdosing

89. How much relief do psychedelics provide you for your headaches / migraines?

- 0% (No Relief)
- 25%
- 50%
- 75%
- 100% (Complete Relief)

SECTION HEADER: TRAUMATIC BRAIN INJURY

ONLY FOR THOSE THAT IDENTIFY TRAUMATIC BRAIN INJURY AS REASON FOR PSYCHEDELIC USE (Q61)

We'd now like to ask you a few questions about your use of psychedelics to treat traumatic brain injury (TBI).

90. What was the primary cause of your traumatic brain injury?

- Sport/recreational activities
- Fall
- Motor vehicle accident
- Blast or explosive injury
- Substance use
- Medical condition (e.g., stroke, tumor, aneurysm)
- Combat-related
- Other, please specify:

91. Which of the following has been most effective in treating your traumatic brain injury?

- *If the answer to Question 18 = "Yes", then it will be shown again here.*
- None - no psychedelic has been effective in treating my traumatic Brain Injury / head traumas

92. What specific symptoms associated with traumatic brain injury are you treating with psychedelics? Check all that apply.

- Mood changes or mood swings
- Anxiety / depression
- Cognitive functioning (e.g., memory, attention, concentration)
- Stress management
- Connection with others
- Headaches / migraines
- Nausea / vomiting
- Dizziness / loss of balance
- Convulsions / seizures
- Sleepiness
- Insomnia / difficulty sleeping
- Other, please specify:

IF ANYTHING EXCEPT "NONE" IN Q91

93. How effective were psychedelics in relieving your traumatic brain injury symptoms / conditions?

INSERT SLIDING SCALE, 0-100

0% - not at all effective 50% - somewhat effective 100% - very effective

(Place a mark on the scale above)

SECTION HEADER: ATTENTION DEFICIT HYPERACTIVITY DISORDER (ADHD)

ONLY FOR THOSE THAT IDENTIFY ADHD AS REASON FOR PSYCHEDELIC USE (Q65)

We'd now like to ask you a few questions about your use of psychedelics to treat ADHD.

94. Which of the following has been most effective in treating your Attention Deficit Hyperactivity Disorder (ADHD)?

- *If the answer to Question 18 = "Yes", then it will be shown again here.*
- None - no psychedelic has been effective in treating my Attention Deficit Hyperactivity Disorder (ADHD)

95. What specific symptoms associated with Attention Deficit Hyperactivity Disorder (ADHD) are you treating with psychedelics? Please check all that apply.

- Mood changes or mood swings
- Anxiety / depression
- Cognitive functioning (e.g., memory, attention, concentration, forgetfulness)
- Stress management
- Connection with others
- Fidgeting / excessive movement
- Excessive talking / interrupting conversations
- Other, please specify:

IF ANYTHING EXCEPT "NONE" IN Q94

96. How effective were psychedelics in relieving your Attention Deficit Hyperactivity Disorder (ADHD) symptoms / conditions?
INSERT SLIDING SCALE, 0-100

0% - not at all effective 50% - somewhat effective 100% - very effective

(Place a mark on the scale above)

SECTION HEADER: PSYCHEDELIC ACCESS AND AFFORDABILITY

We'd now like to ask you a few questions about psychedelic access and affordability.

97. How much money do you typically spend per month on psychedelics, psychedelic use, or psychedelic-assisted therapy?

- $0 - I typically don't spend anything on psychedelics
- $1-99
- $100-249
- $250-499
- $500-749
- $750-999
- $1000-1499
- Over $1500 per month

98. Do you receive financial assistance to help cover the cost of psychedelic treatments by a therapist or health care professional?

- Yes
- No

IF "YES" TO Q98:
98a. Which of the following provide financial assistance for the cost of psychedelic treatments by a therapist or health care professional? Check all that apply.

- Public insurance provider
- Private insurance provider
- Other, please specify:

IF "PRIVATE" INSURANCE PROVIDER IN Q95a:

98b. Please provide the name of your private insurance provider:

____

SECTION HEADER: PSYCHEDELICS AND OTHER SUBSTANCE USE

We'd now like to ask you a few questions about how your use of psychedelics has impacted your use of other substances.

99. Did your use of any of the substances listed below **CHANGE** as a result of your use of psychedelics?
Answer options: CEASED use of the substance completely, DECREASED use of substance, NO CHANGE in use of substance, INCREASED use of substance, INITIATED use of this substance, N/A - I don't use this substance

If the answer to Question 11 = "Yes", then it will make the relevant answer visible.

a. Alcohol
b. Amphetamines / Methamphetamine (e.g., crystal meth, Adderall, Ritalin)
c. Antidepressants (e.g., SSRIs, SNRIs, etc.)
d. Benzodiazepines (e.g., Valium, Ativan, etc.)
e. Cannabis
f. Cocaine / Crack
g. Non-prescription opioids (e.g., heroin, fentanyl, etc.)
h. Prescription opioids (e.g., oxycodone, hydromorphone, morphine, etc.)

i. Tobacco / nicotine

FOR Q100-Q102: IF CEASED OR DECREASED ANY OF THE SUBSTANCES IN Q99

100. Which one of the following psychedelics did you find particularly impactful in **CEASING OR DECREASING** your use of other substances?

- If the answer to Question 18 = "Yes", then it will be shown again here..
- None of the above

101. How have psychedelics helped you to **CEASE OR DECREASE** the use of other substances? Check all that apply.

- They reduced cravings / urges
- They reduced withdrawal
- They made me less anxious or depressed
- They helped me resolve past trauma
- They made me feel more connected with nature
- They made me feel more connected with myself
- They made me feel more connected with others
- They made me feel more connected with spirit
- They changed my relationship with or perspective on other substances
- None of the above

102. How long does the **DECREASE** in substance use typically persist after using psychedelics?

- Less than one week
- 1-4 weeks
- 5-11 weeks
- 12-26 weeks
- >26 weeks
- No set pattern / depends on the substance and circumstances

---

SECTION HEADER: PSYCHEDELICS AND TOBACCO / NICOTINE USE

ONLY IF "YES" TO LIFETIME TOBACCO/NICOTINE USE ON THE FIRST SUBSTANCE USE SECTION (P.6 Q11)

We'd now like to ask you a few questions related to your mood and tobacco / nicotine use, both before and after you used psychedelics.

103. **PRIOR to ever using psychedelics**, in a typical week, how much were you bothered

by…
scale from "1 = Not at all" to "7 = Extremely"

a. Feeling upset
b. Thinking about food a lot
c. Wanting to smoke
d. Troubled sleep
e. Feeling restless
f. Having trouble concentrating
g. Feeling unhappy
h. Eating a lot
i. Having urges to smoke
j. Feeling tired
k. Having trouble sitting still
l. Having trouble paying attention
m. Feeling stressed
n. Feeling hungry
o. Thinking about smoking
p. Waking frequently during the night
q. Feeling fidgety
r. Having trouble thinking clearly
s. Feeling angry

104. **AFTER ever having used psychedelics**, in a typical week, how much were you bothered by…
scale from "1 = Not at all" to "7 = Extremely"

a. Feeling upset
b. Thinking about food a lot
c. Wanting to smoke
d. Troubled sleep
e. Feeling restless
f. Having trouble concentrating
g. Feeling unhappy
h. Eating a lot
i. Having urges to smoke
j. Feeling tired
k. Having trouble sitting still
l. Having trouble paying attention
m. Feeling stressed
n. Feeling hungry
o. Thinking about smoking
p. Waking frequently during the night
q. Feeling fidgety

r. Having trouble thinking clearly
s. Feeling angry

SECTION HEADER: PSYCHEDELICS AND WELLNESS ROUTINES

We would now like to ask you a few questions regarding your psychedelic use and wellness routine.

105. Do you frequently pair your psychedelic use with any other wellness practices or routines (e.g., meditation, stretching, a walk, a bath, etc.)?

- Yes
- No

IF YES TO Q105 display 106-108

106. Which practices do you pair with psychedelic use?
Answer options: Do not pair with psychedelics, Rarely, Sometimes, Often, Always or almost always pair with psychedelics

a. Mindfulness or meditation
b. Mindful movement (e.g., Yoga, Tai Chi, Qi Gong, etc.)
c. Hygiene / spa practices (e.g., bathing, sauna, steam, etc.)
d. Massage or other treatment
e. Physical activity
f. Time in nature

OPTIONS IN 107 SHOULD BE RESTRICTED TO ANSWERS FROM 106

107. Which of the following do you MOST FREQUENTLY pair with psychedelics?

- Mindfulness or meditation
- Mindful movement (e.g., Yoga, Tai Chi, Qi Gong)
- Hygiene / spa practices (e.g. bathing, sauna, steam, etc.)
- Massage or other treatment
- Physical activity
- Time in nature

108 SHOULD SHOW ONLY ONE PRACTICE FROM 107

108. How helpful is the combination of psychedelics and the following practice at improving your well-being?

- Not at all helpful
- Slightly helpful
- Moderately helpful
- Very helpful
- Extremely helpful

SECTION HEADER: SURVEY CLOSE

109. In closing, is there anything else you'd like to share with us about your use of psychedelics?

- Yes
- No

109a. If you answered 'yes', please share your comment here:

________________INSERT LARGE TEXT BOX_________________________

____

Thank you so much for sharing your experiences with us by filling out the Global Psychedelic Survey – English 2023!

Separate conditional activity: Email entry for Amazon gift card draw

Please provide valid email contact information if you'd like to be entered into the draw for 1 of 3 prizes of $500 Amazon gift certificates.

IF YOUR EMAIL IS CHOSEN DURING THE DRAW, WE WILL CONTACT YOU AT THIS EMAIL ADDRESS AND ASK YOU TO "PLEASE PROVIDE US WITH THE MAGIC WORD TO RECEIVE YOUR GIFT CERTIFICATE". PLEASE RESPOND BY RETURNING THE EMAIL WITH THE WORD **MUSHROOM** INSERTED IN THE HEADER OR BODY OF THE TEXT. WE WILL THEN FORWARD YOU THE LINK TO THE GIFT CERTIFICATE.

Please be assured that following the draw, all email addresses will be permanently deleted, and that your complete anonymity will be maintained.

—---------------------Text entry box—----------------------

____

Thank you so much for sharing your experiences with us by filling out the Global Psychedelic Survey – English 2023!
