# Supplementary figures and images for: Psychedelic substitution: altered substance use patterns following psychedelic use in a global survey
Source: Front Psychiatry. 2024 Feb 22;15:1349565. doi: 10.3389/fpsyt.2024.1349565 (PMC10917882; doi:10.3389/fpsyt.2024.1349565)

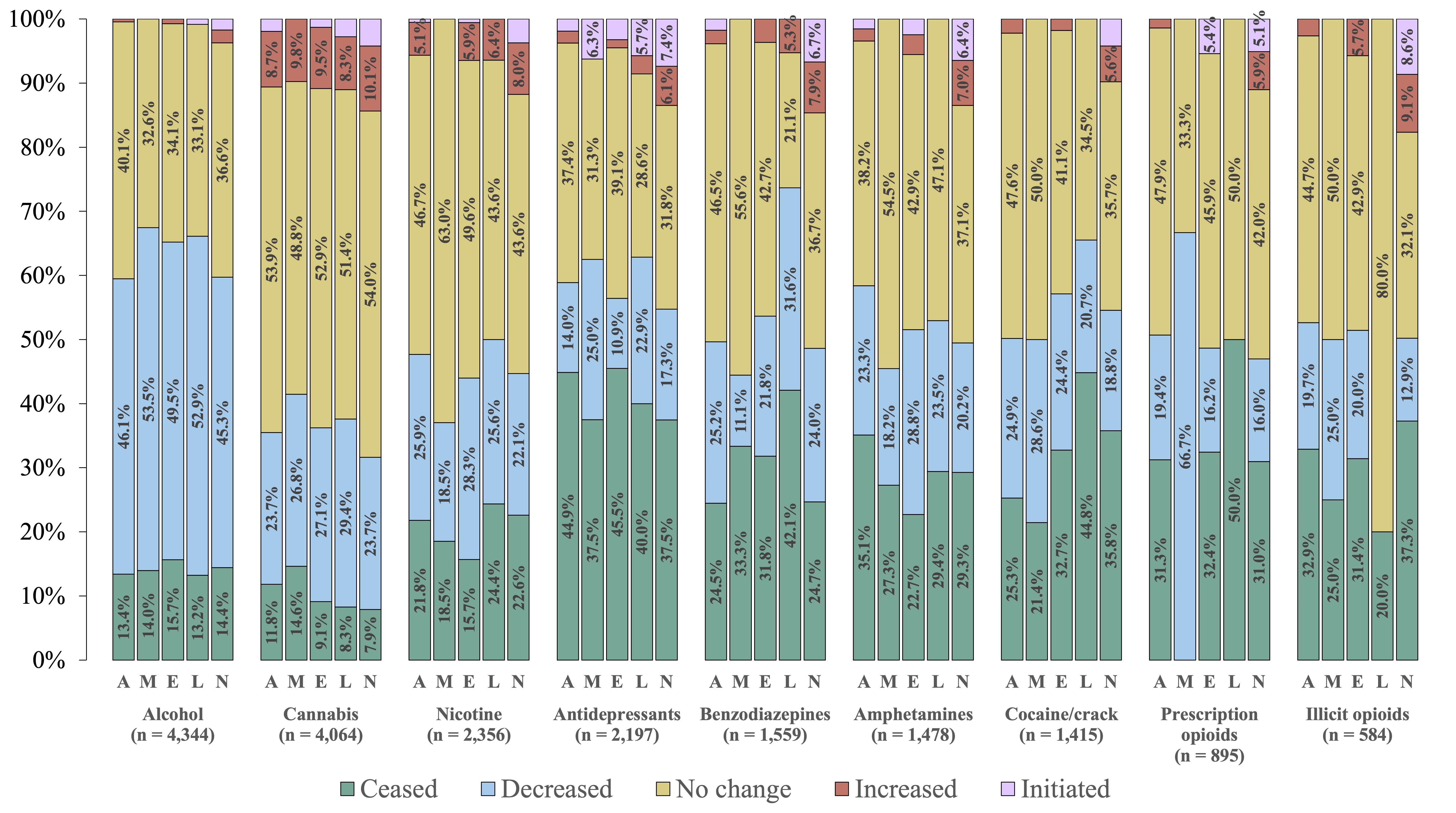

Supplement: Supplementary Figure 1 — Self-reported changes in substance use following psychedelic use among global regions. The number of participants who reported past or current use of each of the substances is listed below each substance. The results are separated based on current primary residence with A = Asia Pacific, M = Central Asia, Middle East, and Africa, E = Europe, L = Latin America, N = North America. Proportions for each category are listed in their respective locations, and values less than 5.0% are not shown. [file Image_1.jpeg]
